# Supplementary material for: Determinants of Successful Aging in a Cohort of Filipino Women
Source: Geriatrics (Basel). 2019 Jan 11;4(1):12. doi: 10.3390/geriatrics4010012 (PMC6473400; doi:10.3390/geriatrics4010012)
Supplement: Supplementary file 1 [file geriatrics-04-00012-s001.pdf]

Supplemental Tables

Supplemental table 1

Age-stratified univariate associations of sociodemographic and behavioral determinants of SA

	Under 60y				60y and over			
	Physiological β (95% CI)	Mental Health β (95% CI)	Cognitive β (95% CI)	Sociological β (95% CI)	Physiological β (95% CI)	Mental Health β (95% CI)	Cognitive β (95% CI)	Sociological β (95% CI)
Age (y)								
<50	Ref	Ref	Ref	Ref				
50-55	-0.05 [-0.28, 0.17]	0.19 [-0.17, 0.54]	-0.11 [-0.54, 0.33]	-0.24 [-0.47,-0.01]				
55-60	-0.11 [-0.34, 0.11]	0.12 [-0.23, 0.47]	-0.09 [-0.52, 0.34]	-0.29 [-0.52,-0.07]				
60-65					Ref	Ref	Ref	Ref
>65					-0.34 [-0.53,-0.16]	-0.29 [-0.51,-0.08]	-0.70 [-0.97,-0.43]	-0.15 [-0.30,0.00]
Marital Status								
Never	0.35 [-0.39, 1.10]	-0.47 [-1.60, 0.67]	0.71 [-0.68, 2.10]	-0.13 [-0.88, 0.62]	-[a]	-[a]	-[a]	-[a]
Married	Ref	Ref	Ref	Ref	Ref	Ref	Ref	Ref
Widowed	0.01 [-0.13, 0.15]	-0.15 [-0.37, 0.08]	-0.37 [-0.64,-0.10]	-0.00 [-0.15, 0.14]	-0.30 [-0.49,-0.11]	0.04 [-0.19, 0.26]	-0.47 [-0.76,-0.19]	-0.03 [-0.19,0.12]
Separated	-0.01 [-0.23, 0.2]	-0.06 [-0.39, 0.27]	0.21 [-0.20, 0.62]	-0.14 [-0.36, 0.08]	-0.09 [-0.64, 0.45]	-0.28 [-0.91, 0.35]	0.37 [-0.44, 1.20]	0.33 [-0.11,0.77]
Educational attainment								
<Primary	Ref	Ref	Ref	Ref	Ref	Ref	Ref	Ref
Primary	-0.03 [-0.17, 0.11]	-0.03 [-0.24, 0.19]	0.97 [0.74, 1.20]	0.18 [0.04, 0.32]	-0.06 [-0.30, 0.19]	0.09 [-0.19, 0.36]	1.00 [0.69, 1.30]	0.22 [0.03, 0.42]
Some secondary	-0.10 [-0.23, 0.04]	0.07 [-0.14, 0.27]	1.60 [1.30, 1.80]	0.17 [0.03, 0.30]	0.09 [-0.15, 0.33]	0.38 [0.11, 0.65]	1.60 [1.30, 1.90]	0.17 [-0.02, 0.36]
Secondary or more	-0.04 [-0.26, 0.17]	0.37 [0.03, 0.70]	2.00 [1.60, 2.40]	0.17 [-0.05, 0.39]	0.08 [-0.22, 0.37]	0.68 [0.34, 1.00]	2.30 [1.90, 2.70]	0.26 [0.03, 0.50]
Urbanicity								
Lowest tertile	Ref	Ref	Ref	Ref	Ref	Ref	Ref	Ref
Mid tertile	-0.21 [-0.35,-0.08]	0.01 [-0.20, 0.23]	0.18 [-0.08, 0.44]	-0.08 [-0.22, 0.06]	-0.25 [-0.48,-0.02]	0.05 [-0.22, 0.31]	0.40 [0.06, 0.74]	-0.05 [-0.24, 0.13]
Highest tertile	-0.29 [-0.42,-0.15]	0.13 [-0.08, 0.34]	0.11 [-0.15, 0.37]	0.05 [-0.09, 0.18]	-0.38 [-0.61,-0.15]	0.02 [-0.25, 0.28]	0.55 [0.21, 0.89]	0.01 [-0.17, 0.19]
Urban SES								
Lowest tertile	Ref	Ref	Ref	Ref	Ref	Ref	Ref	Ref
Mid tertile	-0.02 [-0.17, 0.14]	0.05 [-0.20, 0.30]	0.74 [0.44, 1.00]	0.12 [-0.05, 0.28]	0.02 [-0.24, 0.28]	0.09 [-0.21, 0.38]	0.40 [0.05, 0.76]	-0.08 [-0.29, 0.12]
Highest tertile	-0.07 [-0.23, 0.08]	0.08 [-0.16, 0.31]	1.00 [0.73, 1.30]	0.15 [0.00, 0.31]	-0.01 [-0.26, 0.25]	0.12 [-0.17, 0.41]	1.60 [1.20, 1.90]	-0.01 [-0.20, 0.20]
Rural SES								
Lowest tertile	Ref	Ref	Ref	Ref	Ref	Ref	Ref	Ref
Mid tertile	-0.06 [-0.22, 0.10]	0.13 [-0.12, 0.38]	0.13 [-0.18, 0.44]	0.23 [0.07, 0.40]	-0.07 [-0.36, 0.21]	0.08 [-0.25, 0.41]	0.40 [0.00, 0.81]	-0.02 [-0.25, 0.20]
Highest	-0.07 [-0.22, 0.09]	0.10 [-0.14, 0.34]	0.40 [0.10, 0.70]	0.15 [-0.01, 0.30]	0.14 [-0.13, 0.41]	0.22 [-0.10, 0.53]	1.30 [0.95, 1.70]	0.00 [-0.22, 0.22]

tertile								
Household Composition								
Single person	0.25 [-0.23, 0.73]	0.14 [-0.61, 0.89]	-0.42 [-1.30, 0.50]	-0.25[-0.73, 0.23]	-0.93 [-1.50,-0.33]	-0.17 [-0.86, 0.51]	-0.50 [-1.40, 0.39]	0.16 [-0.31, 0.63]
One nuclear family	Ref	Ref	Ref	Ref	Ref	Ref	Ref	Ref
Horiz. and/or vert. ext. family	-0.07 [-0.21, 0.08]	0.05 [-0.18, 0.28]	-0.14 [-0.43, 0.14]	0.47 [0.32, 0.61]	-0.24 [-0.50, 0.02]	0.07 [-0.23, 0.37]	-0.01 [-0.39, 0.38]	0.34 [0.14, 0.55]
Multi-nuclear family	-0.08 [-0.20, 0.05]	-0.08 [-0.27, 0.11]	0.13 [-0.11, 0.36]	0.32 [0.19, 0.44]	0.01 [-0.22, 0.24]	0.09 [-0.17, 0.36]	0.25 [-0.09, 0.59]	0.22 [0.04, 0.40]
Smoking Status	-0.03 [-0.14, 0.09]	0.10 [-0.08, 0.27]	-0.16 [-0.38, 0.06]	-0.10 [-0.22, 0.02]	0.01 [-0.19, 0.20]	0.14 [-0.09, 0.37]	-0.37 [-0.67,-0.08]	0.06 [-0.10, 0.22]
Alcohol Consumer	0.01 [-0.09, 0.12]	-0.01 [-0.18, 0.15]	-0.02 [-0.22, 0.18]	0.10 [-0.01, 0.21]	0.07 [-0.12, 0.26]	0.09 [-0.12, 0.30]	0.10 [-0.17, 0.38]	0.12 [-0.02, 0.27]
Employed	0.13 [0.02, 0.25]	0.28 [0.10, 0.46]	0.11 [-0.12, 0.33]	- [b]	0.33 [0.14, 0.52]	0.29 [.08, 0.50]	-0.23 [-0.51, 0.05]	- [b]
Insured	0.02 [-0.08, 0.13]	0.11 [-0.06, 0.27]	0.56 [0.36, 0.76]	0.15 [0.04, 0.25]	0.12 [-0.09, 0.33]	0.15 [-0.09, 0.38]	0.38 [0.08, 0.69]	0.08 [-0.08, 0.25]

Note. [a] No observations for never married participants over age 60y.
[b] Not analyzed due to inclusion of employment in construction of sociological domain variable.

Supplemental table 2

Age-stratified multivariable associations of sociodemographic and behavioral determinants of SA

	Under 60y				60y and over			
	Physiological	Mental Health	Cognitive	Sociological	Physiological	Mental Health	Cognitive	Sociological
Variables	β (95% CI)	β (95% CI)	β (95% CI)	β (95% CI)	β (95% CI)	β (95% CI)	β (95% CI)	β (95% CI)
Age (y)								
<50 (ref)								
50-55	-0.05 [-0.27,0.18]	0.18 [-0.17,0.53]	-0.25 [-0.64,0.13]	-0.23 [-0.45,-0.01]				
55-60	-0.11 [-0.33,0.12]	0.09 [-0.26,0.44]	-0.24 [-0.62,0.15]	-0.31 [-0.53,-0.09]				
60-65 (ref)								
>65					-0.30 [-0.50,-0.11]	-0.25 [-0.48,-0.03]	-0.43 [-0.67,-0.18]	-0.15 [-0.31,0.00]
Marital Status								
Never	0.27 [-0.49,1.04]	-0.53 [-1.72,0.66]	0.68 [-0.62,1.98]	-0.01 [-0.76,0.74]	-[a]	-[a]	-[a]	-[a]
Married (ref)								
Widowed	0.02 [-0.13,0.18]	-0.14 [-0.38,0.10]	-0.15 [-0.41,0.11]	-0.01 [-0.16,0.14]	-0.15 [-0.35,0.05]	0.14 [-0.09,0.37]	-0.13 [-0.38,0.13]	-0.05 [-0.21,0.11]
Separated	-0.01 [-0.23,0.20]	-0.08 [-0.41,0.26]	0.2 [-0.18,0.57]	-0.11 [-0.33,0.10]	0.05 [-0.50,0.60]	-0.47 [-1.11,0.16]	0.07 [-0.65,0.79]	0.17 [-0.27,0.62]
Educational attainment								
<Primary (ref)								
Primary	-0.01 [-0.15,0.13]	-0.05 [-0.27,0.17]	0.88 [0.64,1.13]	0.18 [0.04,0.32]	-0.05 [-0.30,0.20]	0.11 [-0.18,0.39]	0.81 [0.48,1.13]	0.25 [0.05,0.45]
Some secondary	-0.05 [-0.19,0.08]	0.06 [-0.16,0.28]	1.45 [1.21,1.69]	0.14 [0.01,0.28]	0.11 [-0.15,0.38]	0.47 [0.16,0.77]	1.26 [0.92,1.60]	0.2 [-0.02,0.41]
Secondary or more	0.04 [-0.20,0.27]	0.35 [-0.01,0.71]	1.75 [1.35,2.15]	0.18 [-0.05,0.41]	0.18 [-0.16,0.51]	0.88 [0.49,1.26]	1.79 [1.37,2.22]	0.33 [0.06,0.60]
Urbanicity								
Low tertile (ref)								
Mid tertile	-0.22 [-0.36,-0.07]	0.02 [-0.20,0.24]	-0.12 [-0.36,0.13]	-0.15 [-0.29,-0.01]	-0.22 [-0.46,0.03]	-0.06 [-0.35,0.22]	-0.08 [-0.40,0.24]	-0.12 [-0.32,0.08]
High tertile	-0.29 [-0.43,-0.15]	0.14 [-0.08,0.35]	-0.21 [-0.45,0.02]	-0.03 [-0.17,0.10]	-0.33 [-0.59,-0.08]	-0.07 [-0.37,0.23]	-0.02 [-0.36,0.31]	-0.09 [-0.29,0.12]
Urban SES								
Low tertile (ref)								
Mid tertile	0.08 [-0.11,0.27]	-0.01 [-0.31,0.29]	0.73 [0.40,1.05]	0.07 [-0.12,0.25]	-0.14 [-0.47,0.19]	-0.09 [-0.48,0.30]	-0.04 [-0.48,0.39]	-0.12 [-0.39,0.15]
High tertile	0.07 [-0.17,0.31]	-0.03 [-0.40,0.35]	0.91 [0.50,1.32]	0.16 [-0.07,0.40]	-0.31 [-0.73,0.12]	-0.33 [-0.82,0.16]	0.31 [-0.24,0.86]	-0.1 [-0.43,0.24]
Rural SES								
Low tertile								

(ref)								
Mid tertile	-0.1 [-0.29,0.10]	0.13 [-0.17,0.44]	-0.34 [-0.67,-0.01]	0.12 [-0.07,0.31]	-0.05 [-0.40,0.30]	0.1 [-0.30,0.51]	0.06 [-0.39,0.52]	-0.01 [-0.30,0.27]
High tertile	-0.13 [-0.37,0.11]	0.09 [-0.28,0.46]	-0.52 [-0.93,-0.12]	-0.07 [-0.30,0.16]	0.16 [-0.26,0.58]	0.25 [-0.24,0.74]	0.48 [-0.07,1.03]	-0.05 [-0.39,0.29]
Household Composition								
Single person	0.17 [-0.34,0.68]	0.26 [-0.53,1.06]	-0.31 [-1.18,0.57]	-0.19 [-0.69,0.31]	-0.88 [-1.49,-0.27]	-0.14 [-0.84,0.56]	0 [-0.80,0.79]	0.19 [-0.30,0.68]
One nuclear family (ref)								
Horizontally and/or vertically extended family	-0.05 [-0.20,0.10]	0.03 [-0.20,0.27]	-0.03 [-0.29,0.23]	0.50 [0.35,0.65]	-0.18 [-0.44,0.07]	0.13 [-0.17,0.43]	0.05 [-0.28,0.39]	0.37 [0.16,0.58]
Multi-nuclear family	-0.03 [-0.16,0.09]	-0.1 [-0.30,0.10]	0.19 [-0.03,0.41]	0.35 [0.22,0.47]	0.1 [-0.13,0.33]	0.14 [-0.13,0.40]	0.06 [-0.23,0.36]	0.22 [0.04,0.41]
Smoking Status	-0.01 [-0.13,0.11]	0.13 [-0.05,0.31]	-0.02 [-0.23,0.18]	-0.14 [-0.25,-0.02]	0 [-0.20,0.20]	0.2 [-0.03,0.44]	-0.08 [-0.34,0.18]	0.09 [-0.07,0.25]
Alcohol Consumer	0.01 [-0.10,0.12]	-0.03 [-0.20,0.13]	0.02 [-0.16,0.21]	0.14 [0.03,0.25]	0.07 [-0.12,0.25]	0.06 [-0.16,0.27]	0.16 [-0.07,0.40]	0.12 [-0.03,0.27]
Employed	0.13 [0.01,0.25]	0.28 [0.09,0.46]	0.22 [0.01,0.43]	- [b]	0.30 [0.11,0.49]	0.32 [0.10,0.54]	-0.01 [-0.25,0.23]	- [b]
Insured	0.02 [-0.09,0.13]	0.06 [-0.12,0.23]	0.26 [0.07,0.45]	0.12 [0.02,0.23]	0.1 [-0.11,0.31]	0.11 [-0.14,0.35]	0.05 [-0.22,0.32]	0.04 [-0.13,0.21]
F	1.43	1.25	12.98***	4.92***	3.29***	2.56***	13.23***	1.89**
R^2	0.0309	0.0271	0.2254	0.0942	0.0981	0.078	0.3114	0.0558

Note. [a] No observations for never married participants over age 60y.
[b] Not analyzed due to inclusion of employment in construction of sociological domain variable.
* p<.05, ** p<.01, *** p<.001
